# The distribution and heterogeneity of excitability in focal epileptic network potentially contribute to the seizure propagation

**DOI:** 10.3389/fpsyt.2023.1137704

**Published:** 2023-03-14

**Authors:** Denggui Fan, Hongyu Wu, Guoming Luan, Qingyun Wang

**Affiliations:** ^1^School of Mathematics and Physics, University of Science and Technology Beijing, Beijing, China; ^2^Epilepsy Center, Sanbo Brain Hospital, Capital Medical University, Beijing, China; ^3^Department of Dynamics and Control, Beihang University, Beijing, China

**Keywords:** focal epilepsy, network excitability, heterogeneity, synchronous seizure propagation, small-world and free-scale network

## Abstract

**Introduction:**

Existing dynamical models can explain the transmigration mechanisms involved in seizures but are limited to a single modality. Combining models with networks can reproduce scaled epileptic dynamics. And the structure and coupling interactions of the network, as well as the heterogeneity of both the node and network activities, may influence the final state of the network model.

**Methods:**

We built a fully connected network with focal nodes prominently interacting and established a timescale separated epileptic network model. The factors affecting epileptic network seizure were explored by varying the connectivity patterns of focal network nodes and modulating the distribution of network excitability.

**Results:**

The whole brain network topology as the brain activity foundation affects the consistent delayed clustering seizure propagation. In addition, the network size and distribution heterogeneity of the focal excitatory nodes can influence seizure frequency. With the increasing of the network size and averaged excitability level of focal network, the seizure period decreases. In contrast, the larger heterogeneity of excitability for focal network nodes can lower the functional activity level (average degree) of focal network. There are also subtle effects of focal network topologies (connection patterns of excitatory nodes) that cannot be ignored along with non-focal nodes.

**Discussion:**

Unraveling the role of excitatory factors in seizure onset and propagation can be used to understand the dynamic mechanisms and neuromodulation of epilepsy, with profound implications for the treatment of epilepsy and even for the understanding of the brain.

## 1. Introduction

Epilepsy is one of the most common neurological disorders, affecting approximately 65–70 million people worldwide (1). Seizures are usually caused by an imbalance of excitatory and inhibitory cortical neuronal cells (2, 3), and are clinically manifested by massive synchronized periodic discharges based on EEG (Electroencephalogram) (4, 5). Neuronal excitability is associated with a variety of factors, such as microbiota (6), proteases, and glial cells (7). These physiological factors influence neuronal gene expression, morphological development, and the cellular activity (8). As factors that directly or indirectly affect the neuronal excitability expression, they are also associated with epileptic seizures (9). Based on the phenomenon of abnormal excitatory-inhibitory imbalances expressed in epilepsy, non-invasive epilepsy treatments often target on modulating the excitability. For example, antiepileptic drugs can reduce the excitability of neurons by acting on ion channels or indirectly acting on ion channels through neurotransmitter receptors (10), and Deep Brain Stimulation(DBS) can produce excitatory or inhibitory fields thus regulating the state imbalance of nerve cells. However, due to the complicated causes of epilepsy, some patients are resistant to the drugs (11), and still requires sufficient theory and practice to refine the stimulation targets and stimulation patterns. In addition, invasive surgical treatment not only requires a delicate preoperative evaluation but also carries the risk of postoperative paralysis, aphasia and even treatment ineffectiveness. Therefore, understanding the triggers of seizures and the mechanisms of neurophysiological rules governing the development of epileptic brain dynamics may provide theoretical support for epilepsy treatment and may even help us to further understand the brain.

The brain is a highly dynamic system, and human thoughts and memories as well as mechanical movements are controlled and operated by this central system of the brain (12). When any of the activity mechanisms within the brain become abnormal or disrupted, the corresponding brain disorders arise. The pyramidal cells of the neuronal cortex receive either excitatory or inhibitory synaptic potentials and generate extracellular currents (13), they will be detected by tools such as EEG or MEG when many continuously arranged neuronal cells discharge together. Epilepsy is caused by a large number of neuronal cells with hyper-synchronous discharges, and the apparently observable switching of electrical signal patterns during seizures has attracted extensive researchers’ attention. The brain is a nonlinear dynamical system, and increasingly mathematical dynamical models have been applied to study and explain the mechanisms of this state transitions (14, 15). For example, models such as Hodgkin-Huxley(HH) (16), Morris-Lecar(ML) (17) elaborate the association of action potential generation with sodium and potassium ions; these describe the behavior of individual neurons at the microscopic level. Models such as Neuron Mass Model (NMM). (18–20) are also included to describe the overall properties of a population of neurons at the macroscopic level, which can better reflect the physiological significance. Typically, a change in the stability of a model caused by a low-dimensional attractor bifurcation in some of the autonomous parameters in the model can induce a seizure-like state of activity. The typical high-frequency rapid discharges (70-120 Hz) that can be recorded at the onset of a seizure and equally accompanied by some low-frequency discharges ( β rhythm and γ rhythm, 20–40 Hz) (21). In some cases, some of the parameters in the model can act as control roles for excitability controlling and can provide a rough depiction of the neural field information in a particular state of the brain, simulating abnormal brain firing. Mature model representations and studies have presented us with some of the mechanisms of brain activity, and therefore such models containing excitability information can be used to study the phenomenon of known epileptic hyperexcitability discharges. Besides, there is a separation of time scales during seizures (22), its recurrent nature also suggesting the existence of a larger time scale of epilepsy such as months, years, etc. This indicates that we cannot ignore the differences and associations between different time-scale variables during our modeling process.

Computational models of epilepsy have rapidly advanced and various dynamic mechanisms within the brain can be revealed through computational models. Due to the diverse pathogenesis of epilepsy, different physiological regions result in similar clinical seizure symptoms (23). From these complex physiological mechanisms, common pathways of epilepsy expression can be identified, and such common pathways involve large brain networks. Simplified dynamical models represent only a single modality, and from a dynamic perspective, structural networks characterizing the connectivity of neuronal circuits are often needed to reflect firing activity close to the real physiological mechanisms. Therefore, a combination of dynamical models and brain networks is required to represent the dynamic evolutionary processes more effectively at the whole brain level. It has been established that different network structures embedded in the model lead to different network states (24, 25), and the overall network structure inevitably affects the pattern of information flow traveling through the network. Brain network as a heterogeneous network, with this pattern also related to the properties of each node, which is supported by the interaction of network structure and node excitability distribution (26). The whole brain structural network seems to be considered in most studies where network factors are analyzed, and subnetworks or local networks are mostly considered for their functionality. It is not clear what role the connectivity patterns or nodal properties within their underlying epileptic networks play in triggering the widespread spread of seizures in focal epilepsy. Therefore, a qualitative analysis of our dynamical models in specific structures is necessary.

In this article, we use the model proposed by Jirsa (27), which is a timescale separated model that can separately simulate different types of epileptic-like seizure signals. We simulated a fully connected network model consisting of 100 nodes, in which highly excitatory nodes are considered as “lesion points,” which convey excitatory information in the brain. Notably, the focal subnetworks of these focal points are connected to each other in a specific connection pattern with prominent connection strength and without disrupting the fully connected form of the original network. We analyzed the effects of these prominently connected focal nodes on the network model under different structures, different degrees of excitability, and different degrees of excitatory heterogeneity, hoping to provide theoretical support for the mechanism of focal epilepsy generation and focal to bilateral seizures.

## 2. Models and methods

### 2.1. Epileptor model

In this paper, we computationally explore the influence factors of seizure propagation of the focal epilepsy network based on the epilepsy oscillator model proposed by Jirsa (27). The model is given as follows:


$${\dot{x}}_{1}=y_{1}-f_{1}\left(x_{1},x_{2}\right)-z+I_{1}$$

$${\dot{y}}_{1}=1-5{\left(x_{1}\right)}^{2}-y_{1}$$

(1) $$\dot{z}=\frac{1}{\tau_{o}}\left(4\left(x_{1}-x_{0}\right)-z\right)\;$$

$${\dot{x}}_{2}=-y_{2}+x_{2}-{\left(x_{2}\right)}^{3}+I_{2}+0.002g\left(x_{1}\right)-0.3\left(z-3.5\right)$$

$${\dot{y}}_{2}=\frac{1}{\tau_{2}}\left(-y_{2}+f\left(x_{1},x_{2}\right)\right)$$

where


$$f_{1}\left(x_{1},x_{2}\right)=\{\begin{array}{c} x_{1}^{3}-3x_{1}^{2}\;\;\;\;\;\;\;\;\;\;\;\;\;\;\;\;\;\;\;\;\;\;\;\;\;\;\;\;\;\;\;\;\;\;\;x_{1}<0\\ \left(x_{2}-0.6{\left(z-4\right)}^{2}x_{1}\right)\;\;\;\;\;\;x_{1}\geq 0\; \end{array}$$

(2) $$f_{2}\left(x_{1},x_{2}\right)=\{\begin{array}{c} 0\;\;\;\;\;\;\;\;\;\;\;\;\;\;\;\;\;\;\;\;\;\;\;\;\;\;\;\;\;\;\;\;\;\;\;x_{1}<-0.25\\ 6\left(x_{2}+0.25\right)\;\;\;\;\;\;\;\;x_{1}\geq -0.25\; \end{array}$$

and

(3) $$g\left(x_{1}\right)=\overset{t}{\underset{t_{0}}{\int }}e^{-\gamma \left(t-\tau \right)}x_{1}\left(\tau \right)dt\;$$

This model includes three groups of variables with different time scales. $x_{1},x_{2}$ are responsible for generating fast oscillations, related to the potential activity of the neuronal membrane, with the shortest time scale. $y_{1},y_{2}$ are responsible for generating SWE (sharp-wave events) and interictal spikes, with a slower time scale $\tau_{2}$ compared to the first group of variables, simulating the membrane potential. The variable z has the largest time scale and represents the slowly varying permittivity variable responsible for guiding the entire system. During epileptic-like seizures, z is associated with slowly changing processes outside the cell, such as ion levels, energy metabolism and oxygen content, etc. In this model, $x_{1}+x_{2}$ can be used to represent the electrographic signatures of a SLE (Seizure like events).

**Figure 1:**
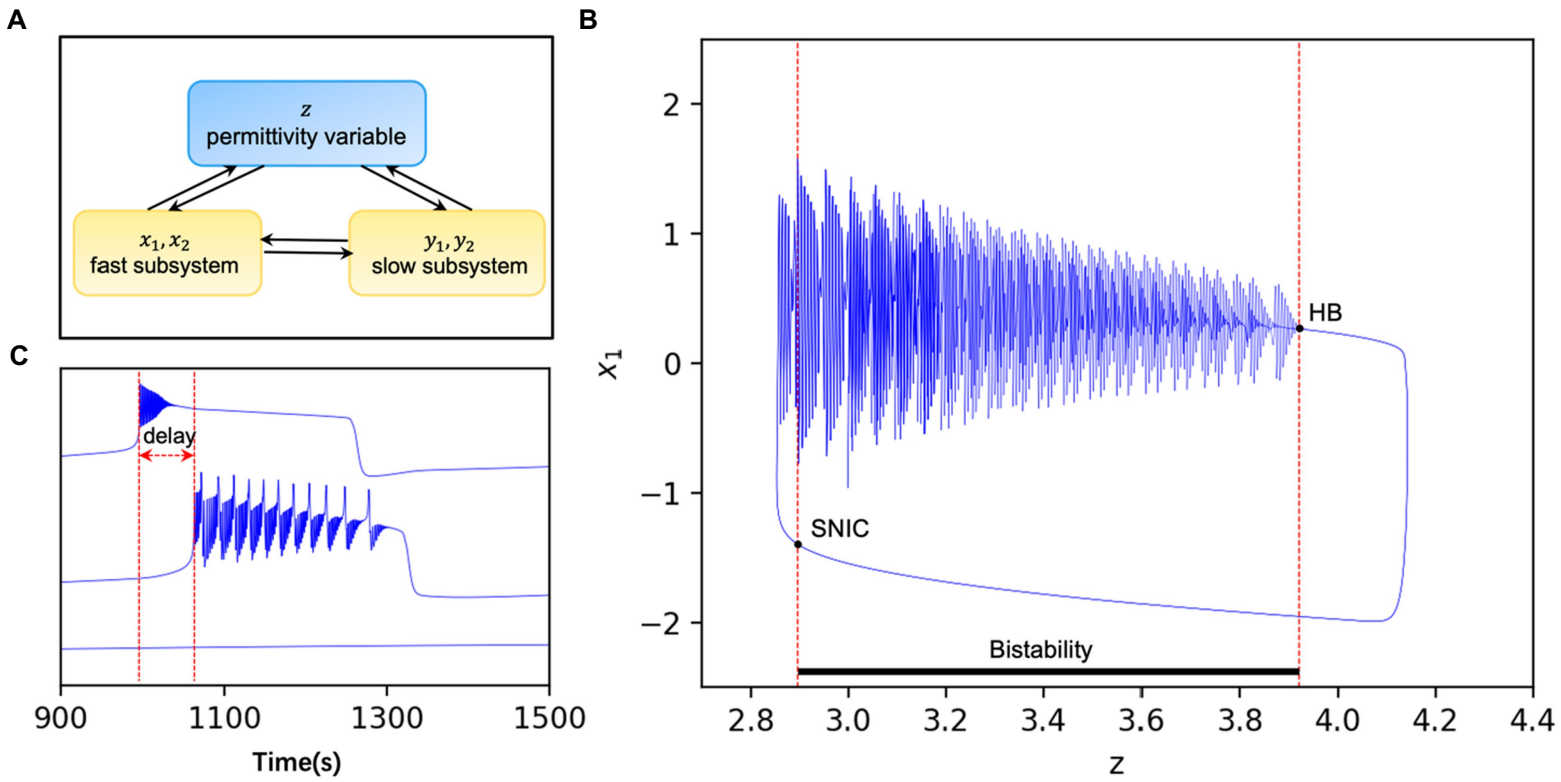
**(A)** Pattern diagram of the model. $x_{1},x_{2}$ are the fast subsystems and $y_{1},y_{2}\;$ are the slow subsystems. In addition to their direct interaction, the two subsystems can also interact with each other indirectly through the extracellular environmental, i.e., the permittivity variable z . **(B)** The system is bistable when the permittivity variable z lies between two bifurcation points and loses stability when the stable limit ring passes through the HB or the stable point passes through SNIC. The whole system varies periodically with time modified from Guo et al. (28). **(C)** Discharges of nodes with different excitability in the same network. When $x_{0}>x_{threshold}$ , the model is in the seizure state and the oscillator will switch periodically between the seizure and interseizure periods (upper panel). When $x_{0}<x_{threshold}$ , the oscillator in the non-ictal state switches between the onset and interictal states due to the perturbations such as network connection and noise, and there is often a time delay with the oscillator in the onset state (middle panel). Also, when $x_{0}<x_{threshold}$ , the model is in the non-seizure state and the oscillator is stable for a long time without perturbations (lower panel).

In addition to the interaction between the fast and slow nervous system in the model, it is also coupled through the permittivity variable z , as shown in Figure 1A (28). The dielectric coefficients are considered to be correlated with excitability and control the onset state of the model. Figure 1B (28) gives a bifurcation diagram of the fast variable $x_{1}$ with regards to the variable *z*. When the dielectric coefficient goes from large to small through the SNIC (Saddle-node on invariant circle), the model transitions from the interictal to the ictal state. Conversely, when the dielectric coefficient goes from small to large through the HB (Homoclinic bifurcation), the model transitions from the ictal state to the interictal state. This bistable mechanism leads to the existence of an “epileptic element” $x_{0}$ in the model that controls state switching, which can be used as the threshold to control the onset of the model (according to current model, $x_{threshold}=-2.05$ ). When $x_{0}<x_{threshold}$ the system stays at a stable fixed point and does not generate seizures, while when the $x_{0}>x_{threshold}$ , the system will transit to the seizure phase. What is more, the excitability of one node depends on the distance between $x_{0}$ and $x_{threshold}$ . The healthy node may also be recruited to present a seizure state under external perturbations if the distance is too close (Figure 1C).

### 2.2. Whole brain network model

To investigate the seizure effects of focal epilepsy on a whole-brain scale, we modeled the brain as a network. Individual brain regions or clusters of neuronal cells can be taken as nodes, and the connections between them are mapped to become the edges of the network. In the dynamical model, each epileptic oscillator can be seen as part of a brain region, and nodal connections can be implemented by coupling in the model.

It has been indicated that fast coupling through synaptic or gap connections does not induce qualitative variations in slow time-scale behavior (29), thus the multi-timescale model of epilepsy with recurrent seizures needs to take into account the slow dielectric coefficients containing cellular parameters (30). This oscillator model is a phenomenological model presenting epilepsy-like activity and has less direct connection to the biophysiological mechanisms embedded in the real human brain. Starting from the phenomenology, the permittivity variable z on the slow time scale is coupled with linear inhibition of the fast and slow subsystems and negative feedback coupling to SLE. In the case of multiple nodes discharging simultaneously, the discharge of node j can be conveyed to the vicinity of node i through axonal transmission, which perturbs the dynamical state of node i . The axonal connections that play the role of axonal transmission are represented in the form of structural connections. In a whole-brain network, all nodes can be coupled using a permittivity variable z that represents a process external to the cell. Thus, a model of a whole-brain network formed by multiple epileptic oscillators is as follows:


$${\dot{x}}_{1,i}=y_{1,i}-f_{1}\left(x_{1,i},x_{2,i}\right)-z_{i}+I_{1}$$

$${\dot{y}}_{1,i}=1-5{\left(x_{1,i}\right)}^{2}-y_{1,i}$$

(4) $${\dot{z}}_{i}=\frac{1}{\tau_{o}}\left(4\left(x_{1,i}-x_{0}\right)-z_{i}-\overset{n}{\underset{j=1}{\sum }}S_{ij}\left(x_{1,i}-x_{1,j}\right)\right)\;$$

$${\dot{x}}_{2,i}=-y_{2.i}+x_{2,i}-{\left(x_{2,i}\right)}^{3}+I_{2}+0.002g\left(x_{2,i}\right)-0.3\left(z_{i}-3.5\right)$$

$${\dot{y}}_{2,i}=\frac{1}{\tau_{2}}\left(-y_{2.i}+f\left(x_{1,i},x_{2,i}\right)\right)$$

where $S_{ij}$ represents the degree of connectivity between individual nodes, which can usually be represented by the structural connectivity matrix of the brain. The model is simulated by fourth order Runge–Kutta, and all parameters in the model are shown in Table 1.

**Table 1:** Model default parameter.

Parameter	Value	Meaning
$I_{1}$	3.1	Current of fast subsystem
$I_{2}$	0.45	Current of slow subsystem
$\tau_{o}$	2,857	Time scale of the permittivity variable
$\tau_{2}$	10	Time scale of the permittivity variable
γ	0.01	Time constant in function g(x)

### 2.3. Network structure and excitatory heterogeneity of seizure nodes

**Figure 2:**
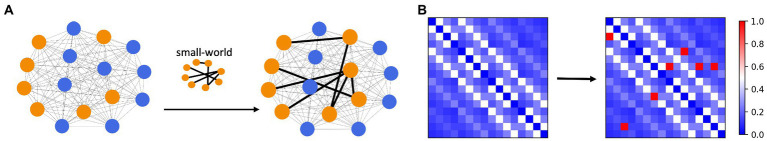
Diagram of network connection structure. **(A)** The strength of connections between nodes in a fully connected network is inversely proportional to the between-node path. The connection pattern between lesion nodes (orange) is topologically specific (small-world connection as an example). The strength of the connection between the lesion nodes was significantly greater than the remaining connections **(B)** The connectivity strength between lesion nodes is extremely prominent, much higher than that between non-lesion nodes, and this matrix can be used as the connectivity matrix *Sij* in the model.

In our work, to explore the seizure propagation of focal epilepsy, we have considered several factors that may influence the outcome of propagation. The first is the connectivity structure of the lesion nodes. Complex network theory provides a rich perspective and tool for brain network studies (31–33), and classical network models such as random networks often have their unique properties that can be used to depict rich brain network connections. Several classical complex network models including random networks, small-world networks, and scale-free networks are introduced into the network dynamics model in this paper. We built a special fully connected network. First, the strength of connections in this network is inversely proportional to the paths between nodes pairs, then the connections between groups of excitatory nodes (which can be considered as lesion nodes) were strengthened to form a specific network model structure individually. In a whole perspective, the network remains a fully connected network with the lesion nodes are prominently connected. This situation can be seen as a special network structure embedded in the original fully connected network, as shown in Figure 2, in which connection strength is of significant differences. In this way, we obtain a connectivity matrix $S_{ij}$ of the fully connected network. Besides, the proportion of focal nodes is also taken into account. Different lesion proportion implies different scales of lesion networks, which is one of the influencing factors that we cannot ignore.

The topological connectivity and the scale of the network can be considered as the “physical properties” of a network, where each node is simulated by a dynamic model, and the variable z in each model represents the degree of excitability of the node, controlled by $x_{0}$ . The difference in excitability of each node in the network can be considered as the unique “intrinsic property” of each network. We replace a set of excitatory nodes with $x_{0}$ following normal distribution into the focal network:

(5) $$\begin{array}{l} x_{0,i}\sim N\left(\mu ,\sigma^{2}\right),x_{0,i}>x_{threshold},\\ for\;i=1,2,\ldots \ldots ,n,i=lesionnode\; \end{array}$$

The excitability and heterogeneity of nodes can be expressed as σ and σ , respectively. The excitatory nodes should not be too close to the threshold and the heterogeneity should not be too large, otherwise some nodes may be included in the non-epileptogenic zone.

## 3. Results

### 3.1. Whole brain network connectivity mechanisms underlying the consistent discharges

**Figure 3:**
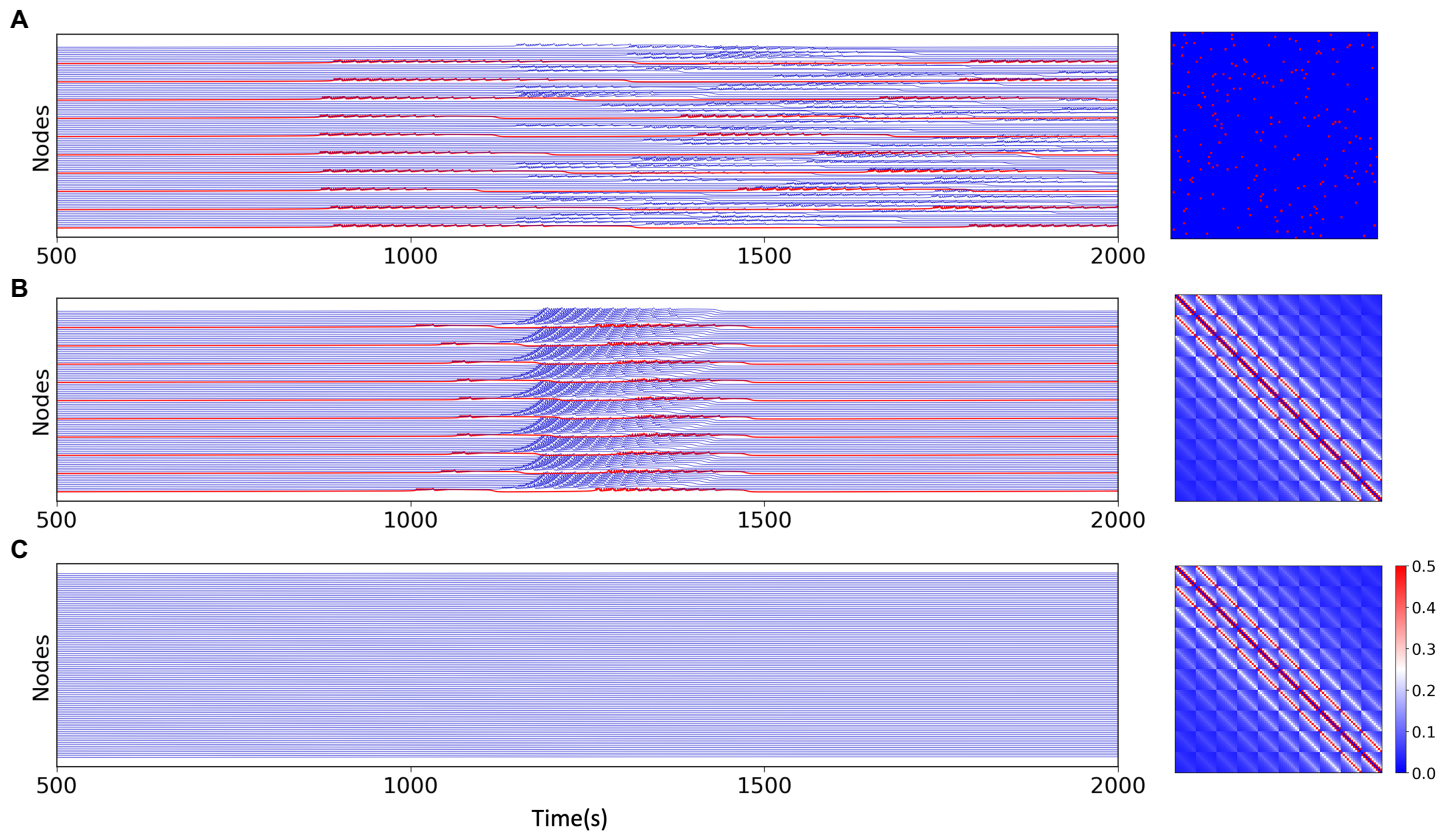
Simulation results for the network model with different network connections and different excitations, the connection matrix is shown on the right. **(A)** Simulation results of the multi-node network model under the random network, where the excitatory nodes (red) can have state switching and a portion of the non-excitatory nodes remain in the stable state. **(B)** Simulation results of the multi-node model with fully connected network. Except for excitatory nodes (red), all non-excitatory nodes generate state switching, which is slightly delayed than excitatory nodes. **(C)** Simulation results of the model without excitatory nodes under fully connected network connections, all nodes do not have state switching.

In the epileptor model, different permittivity coefficients, i.e., variable z , guides the system into different states. And the epileptogenic factor $x_{0}$ included in z can be used as the main parameter to control the degree of excitability of the node. $x_{0}$ located on the left and right sides of the $x_{threshold}$ causes the system to be in a non-oscillatory state and an oscillatory state, respectively, where the oscillatory state can be considered as the seizure state. In a system with individual node, the model is governed by a single excitability index $x_{0}$ . In the network model, the interactions between nodes implies a diversity of node states. This multi-state is not only determined by the initial diverse excitability of the nodes, but the connectivity between nodes embedded in z also influences the state of the nodes in some way. We set some of the nodes in the multi-node network as excitatory nodes and the rest as non-excitatory nodes. We found that when the node network is sparsely connected, due to the presence of excitatory nodes, part of non-excitatory nodes also exhibits state switching, but the overall excitatory synchronization of the network is weak (Figure 3A), but when the node network is fully connected, all the non-excitable nodes are also converted to a “delayed onset” oscillatory state in the network model due to the overlapping of node interactions,and most of the nodes have high excitatory synchronization (Figure 3B). However, without the existence of excitatory nodes, full connectivity between nodes cannot directly cause state switching in some nodes either (Figure 3C). We speculate that the primary condition controlling the dynamical behavior of brain regions or neuronal cells within the brain is their own physiological situation, but the information transfer and interaction relationship between individual units is also a part that cannot be ignored.

### 3.2. The effects of focal network size and excitability patterns on the epileptic seizure periods

**Figure 4:**
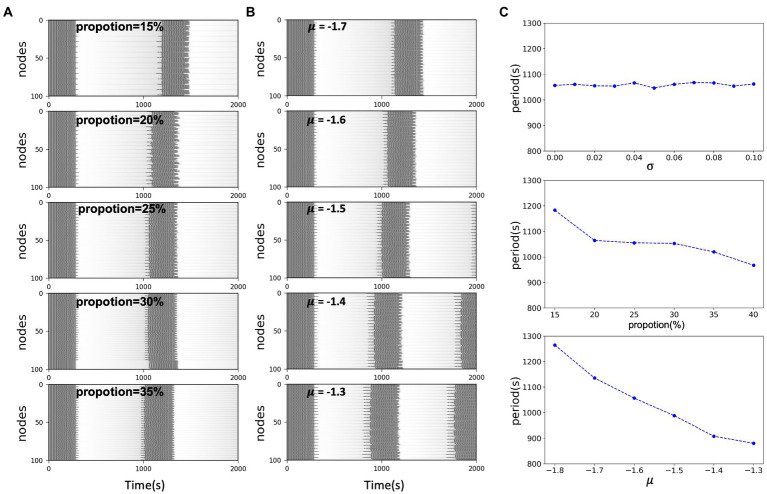
**(A)** Simulated time series of network model with different excitatory node proportions. In the same scale network, the larger the excitability proportion, the shorter the system oscillation period. **(B)** Simulated time series of network model with the same proportion and different excitability level *μ*. In the same scale network, the closer the distance between *x*0 and the threshold, the shorter the oscillation period of the system. **(C)** System oscillation period curve with respect to the excitability heterogeneity of network nodes, network size and the network excitability level, respectively.

Epileptic seizures formally exhibit large-scale periodic coherent discharges. In the results of model simulations, we can also observe periodic changes in the fast and slow subsystems and variable z of excitatory nodes. The multiple time scales involved in epilepsy have been of wide interest, different time scales involve different physiological dynamic behaviors. For epilepsy which may persist with recurrent seizures over a long period of time, time plays an important role, with short periods implying frequent and continuous seizures, which pose a great challenge to the patient himself and to the treatment. Long periods may offer the possibility of interrupting the process of the disease. The electrophysiological mechanisms underlying the switch between ictal and interictal periods in such periodic discharges may conceal the triggering of seizures. In this work, to explore the factors that influence the period of epileptic discharges in a known dynamic background, we considered the network situation in a multi-node model and the excitability of the network nodes. We found that both the proportion of excitatory nodes and their epileptogenic factor $x_{0}$ influence the period of the synchronous oscillation of the nodes. Holding the remaining factors constant, the period of oscillation of the network is negatively correlated with both the averaged $x_{0}$ (i.e., average excitability μ) and the proportion of excitatory nodes. When $x_{0}$ is located in the excitatory region, the greater the distance from the threshold [Figures 4B,C (lower)], the greater the proportion of excitatory nodes [Figures 4A,C (middle)], the shorter the period, the more frequent the state switching of the nodes. And it is not affected by the heterogeneity of node excitability [Figure 4C (upper)]. This may imply that the excitability level of nodes plays an essential role in the network model, and either the change in excitability of a single node itself or the accumulation of multiple similarly excitable nodes will change the overall excitability of the network model, which will be reflected in the periodicity and frequency of seizures.

### 3.3. The secondary effect of focal network topology on its functional activity

**Figure 5:**
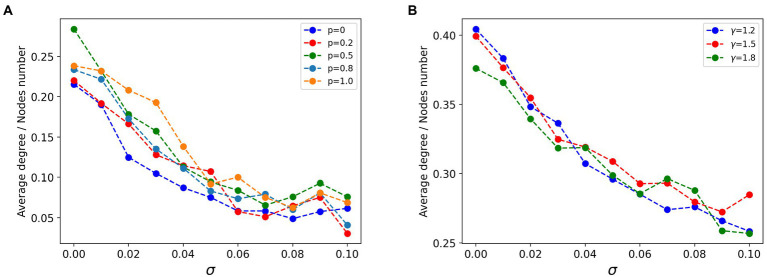
Curves of the relationship between the average degree of lesion node groups and excitatory heterogeneity in the functional network. **(A)** In the strongly connected network of lesion nodes, the higher the node heterogeneity, the smaller the average degree of nodes, and the regular connection (*p* = 0), small world connection (0 < *p* < 1) and random connection (*p* = 1) show the same law. **(B)** In the scale-free strong connection network of focal nodes (*γ* is the power-law exponent of scale-free network), the higher the node heterogeneity, the smaller the average degree of nodes, and the scale-free network structure pattern has little influence.

In the field of brain network research, statistical relationships between signals are often used to build functional networks to investigate the functional coherence of individual brain regions or nodes. As mentioned previously we constructed a fully connected network with lesion nodes specifically connected and strongly connected. After obtaining multiple sets of simulated signals for the same lesion node proportion of the network model, we calculated the Pearson correlation between the signals and used them as the edges of the network to construct a functional network of simulated signals. In this way, we observed the characteristics of the lesion nodes structurally and functionally. We preserved the top 27.5% of the functional connectivity strength to visualize the structure of the functional network. As shown in Figure 5, the average degree of lesion nodes in the functional network correlates with the heterogeneity in the excitability of the lesion nodes, regardless of the connectivity pattern. The functional network after sparing is preserved as strongly connected, with each connection representing a high correlation between signals. When σ = 0, the lesion nodes are homogeneous, and the average degree of all lesion points is maintained around the lesion proportion. Larger σ represents a greater degree of heterogeneity in node excitability, while the potential average activity level (average degree) of the corresponding lesion cluster is negatively correlated with σ , and the connectivity of the lesion cluster becomes smaller as σ increases. However, structural changes in different connectivity patterns under the same type of network did not have a dramatic effect on this trend overall (Figure 5). This implies that excitability in the network remains the dominant factor influencing the state of the system. We noted subtle effects from changes in network structure, but they remained a secondary condition compared to excitability.

## 4. Discussion

Seizures involve abnormalities related to ion channels and synaptic function, and the brain excitation/inhibition circuits develop a dysfunction, which in turn leads to an imbalance of excitation and inhibition in the brain system, usually manifesting as hyperexcitability (34, 35). Some of the disorders caused by excitability-related elemental abnormalities are also accompanied by the generation of epilepsy (36, 37). The process of using DBS for drug-resistant epilepsy is to alter the activity of local field potentials and the excitability of brain networks by remote thalamic stimulation or direct cortical stimulation (38). It is thus clear that excitability is a never-ending subject in the field of epilepsy. However, epilepsy remains a challenge in modern medicine, with its complex temporal and spatial scales, and the seizure mechanisms involved have not been fully revealed. Existing ideas include recording a series of imaging data before and after a clinical seizure, which allows to analyze and predict the seizure and propagation of epilepsy, etc. (39–41). Data analysis is difficult to avoid the specificity brought by individual data, and models can fill the missing part of data analysis. There is a rich electrophysiological mechanism behind the operations of the brain, and some of these transitions can be well reproduced by existing dynamical models, and mature nonlinear dynamical theories can be combined with models to explain some brain-like phenomena. A strong and distinct state switching mechanism exists in epilepsy and is accompanied by a certain periodicity. Combining excitatory factors with computational models to explore the mechanisms of seizures can provide theoretical support for our treatment and study of epilepsy.

In this article, we first analyze the nonlinear dynamical features in the model to explore the mechanisms of abnormal discharges. We found that this abnormal discharge is controlled by a set of bifurcations, with seizures starting at the saddle-point bifurcation and ending at the homozygous bifurcation. The simple model generates abnormal discharges on the premise that to control the model lies within the excitatory region. We also found that excitability is the main factor affecting the model state in a mutually coupled network model. Non-excited nodes have a certain probability to produce a delayed discharge behavior over excited nodes in the case of node coupling overlapping. Such a delayed spontaneous discharge phenomenon helps us to understand the direction of information flow in epileptic networks. In addition to this, we control the distance of the parameters of node excitability and the proportion of excitable nodes in the network, which significantly affect the period of system discharge. This implies that changes in excitability in either degree or extensity affect the system state. The effects of altered system excitability are reflected in both microbiological and macroscopic computational models. Complex systems often contain multi-element interactions, and multi-element excitatory heterogeneity has been similarly shown to play with a role in the propagation of epilepsy when the overall excitability of the network system is constant (42). In our work, the lower the excitability heterogeneity, the stronger the association between clusters of excitatory nodes, which is reflected in the functional network of lesion nodes after model simulation. We suggest that excitability is in a primary position compared to other factors including network coupling and network structure, and that excitability can produce effects on the system in multiple dimensions.

In our whole-brain network, network coupling is not based on structural connectome, but rather a structural network with prominent lesion connections, which theoretically establishes a deeper understanding of the relationship between excitability and the system. The advantage of this is a clearer understanding of the coupling information, and the disadvantage is the lack of physiological information about the real situation of the brain. Currently, in the context of such fully connected networks, there is no good correspondence between simulated signals and known specific network structures for analysis, which is our later effort. It is expected that the dynamic flow of neural information in specific network structures can be revealed. During the model simulation, excitability shows its main role in controlling the state of the system. In the case of epilepsy, such results are inevitable. Therefore, there is a strong need to focus the perspective on potential influences beyond excitability to provide more diverse theoretical support for seizure mechanisms as well as modeling. In conclusion, this study analyzed how excitability parameters in the model affect the dynamic switching as well as the intrinsic properties of the network system in different perspectives by modeling the dynamics and parameter modulation of the epileptic network, reflecting the importance of excitability factors in the epileptic system.

## Data availability statement

The original contributions presented in the study are included in the article/supplementary material, further inquiries can be directed to the corresponding authors.

## Author contributions

DF proposed and supervised the project and contributed to writing the manuscript. HW analyzed the data, performed the experiments, and wrote the manuscript. QW and GL supervised and revised the manuscript. All authors contributed to the article and approved the submitted version.

## Funding

This research was supported by the National Natural Science Foundation of China (Grants 12072021 and 11932003) and the Young Teachers International Exchange Growth Program (QNXM20220049).

## Conflict of interest

The authors declare that the research was conducted in the absence of any commercial or financial relationships that could be construed as a potential conflict of interest.

## Publisher’s note

All claims expressed in this article are solely those of the authors and do not necessarily represent those of their affiliated organizations, or those of the publisher, the editors and the reviewers. Any product that may be evaluated in this article, or claim that may be made by its manufacturer, is not guaranteed or endorsed by the publisher.
